# Disability incidence and functional decline among older adults with major chronic diseases

**DOI:** 10.1186/s12877-019-1348-z

**Published:** 2019-11-21

**Authors:** Joelle H. Fong

**Affiliations:** National University of Singapore, 469C Bukit Timah Road, Singapore, 259771 Singapore

**Keywords:** Aging, Disability incidence, ADL disability, Oldest old, Longitudinal research, G22, H51, H75, C24

## Abstract

**Background:**

More than 80% of elderly Americans have at least one chronic disease. While past studies have shown that hierarchical patterns of functional loss may differ by gender and institutional settings, little is known about whether such patterns differ in relation to chronic health condition. The aim of this study is to investigate the pattern of functional loss among older adults with major chronic illnesses, and to compare their onset and ordering of incident ADL disability with those of persons without such conditions.

**Methods:**

We use a nationally representative sample of persons aged 80+ from the 1998–2014 Asset and Health Dynamics of the Oldest Old survey. The group with major noncommunicable diseases (including cardiovascular disease, cancer, chronic respiratory disease, and diabetes) comprises 3,514,052 subjects, while the comparison group comprises 1,073,263 subjects. Self-reports of having difficulty with six distinct ADLs are used to estimate disability incidence rate. Nonparametric statistical methods are used to derive median onset ages and ADL loss sequence separately for each group.

**Results:**

Older adults with major chronic diseases have higher rates of incident disability across all ADL items. Estimated median onset ages of ADL disabilities for the full sample range from 91.5 to 95.6. Disability occurs earlier for chronically ill persons (onset ages 91.1–95.0) than for those in the comparison group (onset ages 93.5–98.1). Among those with major chronic diseases, the ADL loss sequence ordered by median ages of disability onset is bathing, walking, dressing, toileting, transferring and eating. The activities are also distinctly separated into an early-loss cluster and a late-loss cluster. Although the loss sequence derived for the comparison group is largely similar, disability progression for those with major chronic diseases is compressed within a shorter timeframe and the timing gaps between adjacent disabilities are smaller.

**Conclusions:**

Older Americans with major noncommunicable diseases face an earlier and steeper slope of functional decline. Chronic care delivery programs should adapt to dynamic changes in older patients’ functional status. Health interventions to help patients delay disability onset and optimize functional autonomy within emerging models of chronic care should especially target early-loss activities such as bathing, dressing, and walking.

## Background

The onset of functional disability is a dynamic and progressive process. As one ages, health problems accumulate and people start to lose their ability to perform activities of daily living (ADLs), such as dressing, using the toilet, bathing, and eating. Past studies have shown that the pattern of ADL disability in geriatric populations follows a distinct progression [1–9]. These hierarchical patterns of functional decline or ADL scales – typically established using item response theory (IRT) methods and hazard models – have been reliably and validly assessed not only for the institutionalized population but also more generally for community-dwelling older adults [7]. Several studies have also highlighted that disability progression differ by gender [3]; time periods [6]; institutional settings (e.g. residential setting, nursing home) [6]; types of ADL items [4, 10]; and countries [11, 12]. Yet, little is known whether the pattern of functional loss differs in relation to chronic health condition.

A closer examination of the pattern of functional loss among older persons with major chronic illnesses is valuable for two reasons. First, there is strong theoretical basis that functional disability onset is driven by physiological changes associated with aging and underlying chronic diseases [13]. This notion has been borne out empirically in prior studies which demonstrate strong associations between the increased incidence of functional disability and chronic diseases such as diabetes, stroke and heart disease among the elderly [13–15]. Accordingly, the onset, ordering and general pattern of incident ADL disability among chronically ill persons may be distinct from their healthier peers. Second, chronic diseases are among the most prevalent and costly health conditions in the United States. 85% of Americans over 65 years of age have at least one chronic health condition and 60% have at least two chronic conditions [16]. In particular, cardiovascular diseases (CVD), cancers, chronic respiratory diseases and diabetes impose a disproportionate impact on the overall disease burden. Known as the ‘big four’ noncommunicable diseases (NCDs), these four conditions are the leading causes of disability and death in the United States [17–19].

The purpose of this study is to investigate the pattern of functional loss among older adults with major chronic illnesses, and to compare their onset, ordering and general pattern of incident ADL disability with those of persons without such conditions. Major chronic diseases are defined to include CVD (stroke, heart attack, and heart diseases), cancer, diabetes and chronic lung disease. We use a nationally representative sample of oldest-old adults aged 80 and older from the 1998–2014 Asset and Health Dynamics of the Oldest Old (AHEAD) study. Respondents interviewed at the 1998 baseline are followed across 10 survey waves. We divide respondents into two groups (those who had or developed a major chronic disease during the observation period versus those who did not). For each group, we first report the cumulative incidence rates of disability and then derive age distributions of disability onset by ADL item using nonparametric statistical methods. Median ages of incident disability drawn from the distributions are used to identify the hierarchical ADL loss sequence for those with and without major chronic diseases.

Closest to this present study, Dunlop et al. [3] demonstrate that multiple waves of survey data can be pooled together to evaluate the hierarchy of disability. In that study, the authors used data from the 1984–1990 Longitudinal Study of Aging and found that the ADL loss sequence ordered by median ages of disability onset was: walking, bathing, transferring, dressing, toileting, and eating. Some studies also explore using IRT methods on longitudinal data. One study which applied the Rasch scaling model document an ADL hierarchy of bathing, dressing, transferring, toileting, walking, and eating among adults aged 85 and above from the 1983–96 Aging in Manitoba Longitudinal Study [6]. Also using the Rasch model, Fong and Feng [12] report a somewhat similar sequence (bathing, walking, dressing, toileting, transferring, and eating) based on data from the 1998–2008 Health and Retirement Study. Accordingly, it is useful to assess whether the functional loss sequences derived in this present study are consistent with these documented patterns of functional decline for older Americans.

## Methods

### Data and measures

Data is obtained from the 1998–2014 Asset and Health Dynamics of the Oldest Old study. The AHEAD is a prospective panel study of older Americans born in 1923 or earlier, and respondents are interviewed every 2 years since 1992. We set 1998 as the baseline because ADL question wordings and response coding for AHEAD respondents was made consistent only when the AHEAD merged with the Health and Retirement Study that year [20]. The AHEAD contains detailed information on sociodemographic characteristics, family structure, physical health, cognition, and living arrangements. The 1998 AHEAD survey covered 5951 respondents aged 75 and above; a complete description of the AHEAD is given elsewhere [21, 22]. Our study sample comprises 1604 older adults aged 80+ who have zero ADL disabilities in 1998, and complete health and mortality data for all follow-up waves. This represents about 27% of the 1998 AHEAD cohort. The weighted study population totals 4,587,315.

Functional disability is measured by self-reports of having difficulty with basic self-care tasks. AHEAD respondents are asked: “Because of a health or memory problem do you have any difficulty with [ADL]?, where [ADL] refers to dressing; walking across a room; bathing; eating; getting in and out of bed; and using the toilet.” The responses to each ADL item are coded as six dichotomous variables in each wave [23]. The AHEAD survey also collects information on a set of doctor-diagnosed health problems and chronic conditions, including the ‘big four’ NCDs, in all waves. Respondents are asked, “Has a doctor ever told you that you have had a [chronic condition]?” Those who responded affirmatively to questions relating to heart disease, stroke, cancer, diabetes, and chronic lung disease in 1998 or at any point during the observation period are thus categorized as persons with major chronic illnesses. Based on this classification, the number of respondents with major NCDs is 1203, while the number of respondents without these NCDs is 401.

### Statistical analyses

To determine whether individuals with major chronic conditions are at higher risk of disability onset than their peers, we calculate the proportion of new cases of disability (e.g. bathing) and report the total disability incidence rates by ADLs for each risk group. The weighted population of the group with major chronic illness comprises 3,514,052 subjects (unweighted: 1203), while the comparison group comprises 1,073,263 subjects (unweighted: 401). Death rates by risk group and chronic health status are also evaluated. Discrete-time hazards models are then used to evaluate the age distributions of disability onset and ADL ordering for each group. Specifically, following Dunlop, Hughes, & Manheim [3], we utilize the nonparametric Turnbull [24] algorithm which relies on an iterative procedure to estimate the failure probabilities at discrete time points.

The individual is the unit of analysis. Binary variables are created for each ADL (e.g. bathing) to indicate whether or not a subject developed that disability during the follow-up period. The Turnbull procedure is suitable since the disability data on hand for each subject observed is interval-censored. That is, disability was monitored at 2-year intervals so exact time of disability onset is unknown. Thus, for instance, an 85-year old respondent who has no difficulty bathing in 2000, fails to respond in 2002, then reports needing help with bathing in 2004 will be assigned a bathing disability onset interval of age 85–89. Respondents who do not have that disability over the observation window, or who died prior to disability incidence, are treated as censored. Using the Turnbull survival estimates, we derive the cumulative hazard curve for discrete data (analogous to Kaplan-Meier curves for continuous data) to illustrate age distributions of onset. Median onset ages are used to determine a representative ADL loss sequence for each risk group. Base year individual-level weights are applied in all analyses to derive a nationally representative sample and correct for the oversampling of Hispanics, Blacks, and households in the state of Florida in the survey. Analyses are conducted using STATA, version 14.0 (StataCorp, College Station, Tex, USA).

## Results

### General characteristics of the study population

Table 1 presents the demographic characteristics of the weighted study population. At the 1998 baseline, the mean age of the subjects is 84.3 years and 60% are female. 57% are widowed, 36% are married with spouses alive, and the rest never married. All subjects began with no ADL disabilities at baseline. By the 2006 mid-point, however, many older adults report experiencing difficulty with self-care tasks. About a third of the respondents (34.3%) report difficulty bathing and 30.3% report difficulty walking. Fewer subjects face difficulty dressing (28.4%), transferring from bed/chair (18.3%), and eating (17.6%). Prevalence of ADL disabilities increases over time. By 2014, the proportions in the weighted sample having functional limitations are: 50.0% (bathing), 51.0% (dressing), 33.6% (transferring), 42.7% (walking), 33.6% (toileting), and 36.1% (eating).

**Table 1:** Characteristics of subjects in the weighted AHEAD sample

Variable	Mean
Age as at baseline	84.3 (3.56)
Female	60.0%
Years of education	11.3 (3.48)
Marital status:	
Not married	7.3%
Married	36.2%
Widowed	56.5%
Prevalence of ADL disabilities as at wave 2006:	
Bathing	34.3%
Dressing	28.4%
Transfer bed /chair	18.3%
Walking	30.3%
Toileting	21.1%
Eating	17.6%
Prevalence of ADL disabilities as at wave 2014:	
Bathing	50.0%
Dressing	51.0%
Transferring bed /chair	33.6%
Walking	42.7%
Toileting	33.6%
Eating	36.1%
Ever have a ‘big four’ chronic disease (1998–2014)	76.6%
By condition:	
Cardiovascular disease (CVD)	59.4%
Cancer	22.0%
Diabetes	14.5%
Chronic lung disease	13.0%
Any of these ‘big four’ conditions	75.0%

Notes: The weighted full sample comprises 4,587,315 subjects (unweighted: 1604). Of these, 3,514,052 subjects (unweighted: 1203) either had a major chronic disease at 1998 baseline or developed such a condition over the follow-up period. The comparison group comprises 1,073,263 subjects (unweighted: 401)

About three-quarters of the subjects or 76.6% had or developed at least one ‘big four’ chronic disease over 1998–2014. Of the four chronic conditions, CVD is most prevalent with 59.4% of the weighted sample reporting that they were ever diagnosed by a doctor to have stroke, heart attack or heart disease. This is followed by cancer (22.0%), diabetes (14.5%), and finally, chronic lung disease (13.0%). Not surprisingly, mortality is rather substantial among the oldest-old adults. Approximately 69% of the respondents interviewed at 1998 baseline remained alive as at wave 2002, while 40% of them survived to wave 2006. Overall, 92.8% (1488) died over the 16-year the observation period and 1.8% (29) are lost to follow-up or attrited. The oldest surviving subject is 104.4 years old at 2014 cut-off.

### Disability incidence

Table 2 presents the disability incidence rates for persons with and without major chronic conditions. Cumulative incidence, expressed in percentages, is the number of new cases of a specific ADL disability (e.g. walking) observed over 1998–2014 divided by the size of the subpopulation initially at risk. Results show that those with ‘big four’ NCDs are at higher risk of becoming functionally impaired. Over the 18-year period, the total incidence rates for bathing, dressing walking, transferring, toileting, and eating are respectively 44, 41, 42, 29, 31, and 27% for persons with major NCDs and 36, 33, 32, 25, 25, and 19% for persons without these diseases. It is also evident that chronically ill persons who become disabled experience higher death rates than their non-ill disabled counterparts. This holds systematically across all ADL types. For example, the risk of developing bathing disability *and* being dead by 2014 is 40.6% for a person with major NCDs as compared to only 32.4% for a person without such conditions. Although incidence rates convey information about the risk of becoming disabled, they do not provide insights into the timing or sequence that each disability type occurs over the life course.

**Table 2:** Change in Disability over 18 years (1998 to 2014) among elderly without baseline ADL disabilities

Activity	Incident disability by 2014, %			No Incident disability by 2014, %	
	Alive in 2014	Dead by 2014	Total incidence	Alive in 2014	Dead by 2014
No major chronic condition					
Bathing	3.5%	32.4%	35.8%	4.0%	60.2%
Dressing	3.8%	29.3%	33.1%	3.6%	63.3%
Walking	3.1%	29.0%	32.1%	4.4%	63.6%
Transferring	2.7%	22.6%	25.3%	4.8%	69.9%
Toileting	3.0%	22.3%	25.3%	4.4%	70.3%
Eating	2.1%	17.1%	19.1%	5.4%	75.5%
Have major chronic condition					
Bathing	3.5%	40.6%	44.1%	1.7%	54.2%
Dressing	3.4%	37.3%	40.7%	1.8%	57.5%
Walking	3.2%	38.4%	41.5%	2.0%	56.4%
Transferring	2.6%	26.7%	29.3%	2.6%	68.1%
Toileting	2.4%	28.3%	30.8%	2.8%	66.4%
Eating	2.5%	24.3%	26.8%	2.8%	70.5%

*Notes*: Weighted estimates using baseline individual-level weights. The weighted population of the risk group with major chronic condition over 1998–2014 comprises 3,514,052 subjects (unweighted: 1203), while the comparison risk group comprises 1,073,263 subjects (unweighted: 401). Subjects have no baseline (1998) ADL disabilities and have complete information through 2014 or death

### Principal component analysis

Before proceeding to order the ADLs, it is useful to first ascertain that the six distinct items can be combined to form an ordering. We perform principal component factor analysis to investigate the underlying dimensions of the data. If the responses to the ADL questions are characterized by a single general dimension, then the six ADLs can be meaningfully combined, otherwise not. ADLs that are less scalable with other items should be dropped. The results confirm the existence of a single general dimension across the ADL items in each survey wave used. In wave 2006, for instance, the first principal component explains a high percentage of the total variance (58.4%); the Cronbach alpha value of 0.86 is also relatively high indicating reliable estimates. The individual item-factor loadings on the first component of 0.72–0.79 are well above the threshold of 0.4 [2].

### Age distributions of disability onset

**Fig. 1:**
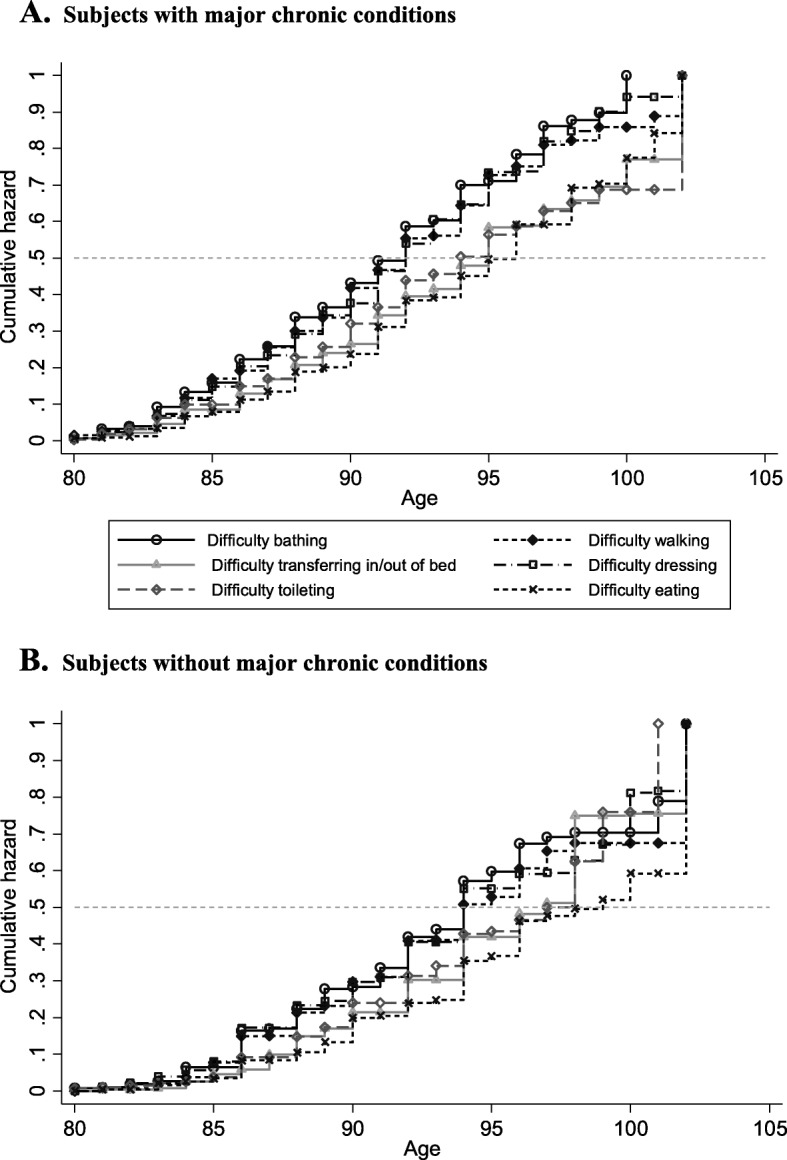
Age distributions of onset by ADL disability. Panel **a** Subjects with major chronic conditions. Panel **b** Subjects without major chronic conditions. *Notes*: Weighted estimates using baseline individual-level weights. The weighted population of the risk group with major chronic condition over 1998–2014 comprises 3,514,052 subjects (unweighted: 1203), while the comparison risk group comprises 1,073,263 subjects (unweighted: 401).

Figure 1 shows the age distribution of onset by activity for each risk group. The cumulative hazard functions from the Turnbull analysis are upward sloping implying that risk of disability onset increases with age. We see a relatively clear separation of individual curves into two clusters. Specifically, the bathing, walking, and dressing curves lies well above the other three curves (toileting, eating and transferring). In other words, the risk of bathing, walking, and dressing disability onset is considerably higher as compared to toileting, eating and transferring disability onset. The small distances between the top three curves, especially for subjects with major chronic conditions, suggest there may not be a strong ordering of these disabilities in the ADL sequence. We note that the risk of eating disability is generally low for both risk groups across all ages values examined.

Table 3 presents the derived median ages of disability onset and interquartile ranges. Results are presented for the entire sample and by chronic health status (*n* = 401 no major chronic condition; *n* = 1203 have major chronic condition). For the full sample, the ordered median ages at disability onset are 91.5 for bathing, 91.8 for dressing, 91.9 for walking, 94.4 for toileting, 94.5 for transferring, and 95.6 for eating. This yields a representative ADL ordering of ‘**BDWTPE**’ (or bathing, dressing, walking, toileting, transferring, and eating). These findings mirror the patterns of disability illustrated in Fig. 1. Specifically, there is an early loss cluster (‘BDW’) and a late-loss cluster (‘TPE’). Median onset ages for bathing, dressing, and walking are extremely close which supports the notion that there is a weak ordering of these three disabilities for older American adults. The ordering between toileting and transferring disabilities is also weak.

**Table 3:** Median Age of Onset by ADL Disability

	All (*N* = 1604)	No major chronic condition (*n* = 401)	Have major chronic condition (*n* = 1203)
ADL disability	Median (25, 75%)^a^	Median (25, 75%)	Median (25, 75%)
Bathe (B)	91.5 (87.2, 95.9)	93.5 (102.2, 97.0)	91.1 (86.8, 95.5)
Dress (D)	91.8 (87.4, 96.6)	93.9 (155.4, 97.9)	91.4 (86.9, 96.0)
Walk (W)	91.9 (87.5, 96.8)	93.6 (88.3, 159.5)	91.5 (87.3, 96.1)
Toilet (T)	94.4 (89.2, 100.0)	97.0 (92.0, 101.2)	93.9 (88.8, 101.2)
Transfer (P)	94.5 (89.7, 99.9)	96.6 (90.8, 101.0)	94.2 (89.4, 100.9)
Eat (E)	95.6 (90.4, 101.1)	98.1 (93.0, 101.4)	95.0 (90.2, 99.7)

^a^25, 75% = interquartile range

*Notes*: The ADL disabilities are presented in ascending order of their median ages of disability onset (rounded to one decimal place). Weighted estimates using baseline individual-level weights. The weighted population of the risk group with major chronic condition over 1998–2014 comprises 3,514,052 subjects (unweighted: 1203), while the comparison risk group comprises 1,073,263 subjects (unweighted: 401). Subjects have no baseline (1998) ADL disabilities and have complete information through 2014 or death

**Fig. 2:**
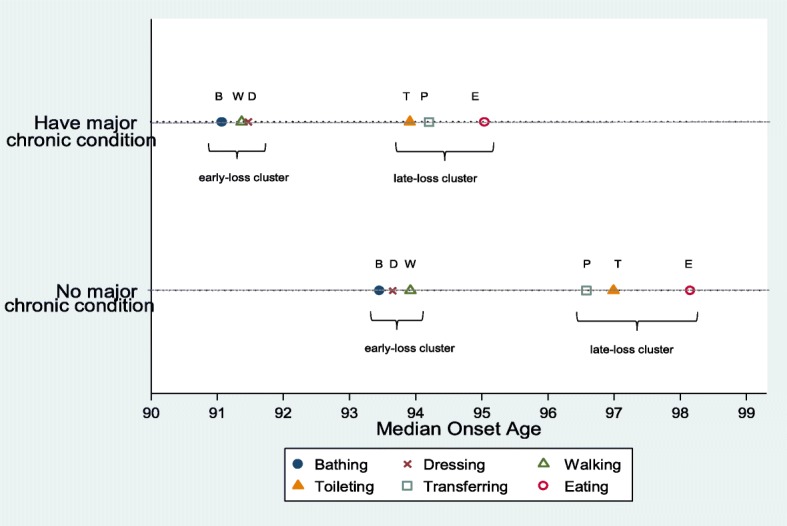
Onset age of ADL disabilities for those with and without major chronic conditions. *Notes*: The weighted population of the risk group with major chronic condition over 1998–2014 comprises 3,514,052 subjects (unweighted: 1203), while the comparison risk group comprises 1,073,263 subjects (unweighted: 401)

Results also indicate that patterns of disability onset and functional decline differ by chronic health status in several ways. First, the activities ordered separately for each risk group reveal a ‘**BDW-TPE**’ sequence for persons suffering from major chronic diseases and a ‘**BWD-PTE**’ sequence for persons without such conditions. Second, the median onset ages are systematically earlier for persons with major NCDs (range 91.1–95.0) than for those without (range 93.5–98.1). This holds across all activities and differences can be substantial. For example, chronically ill individuals experience difficulty using the toilet at age 93.9 on average whereas their counterparts need help for the same activity only about 37 months later at age 97.0. For visualization, a graphical comparison of the summary ADL orderings is provided in Fig. 2. There is evidence that disability onset is compressed within a shorter timeframe for oldest old adults with major NCDs than for those without. For the chronically ill, the total estimated gap between the first and last disability onset is only 3.9 years (compare 4.6 years for those without major chronic conditions).

We also conducted additional analyses stratified by gender. Comparing females with and without major chronic diseases, for example, we find that the patterns of disability for each subgroup reveal an early loss cluster and a late-loss cluster (although the exact sequence of the ADLs may vary between subgroups). The earlier finding that median onset ages are systematically earlier for those with major NCDs also hold when the analyses is conducted separately by gender. For instance, median ADL onset ages for chronically ill females are 90.3–94.0 as compared to 92.6–98.6 for females without the diseases. Consequently, functional decline occurs at a more rapid pace and is more compressed among females (and separately, males) with major NCDs as compared to their same-sex peers.

## Discussion

Disability and functional loss are not static constructs in old age. This paper presents new evidence on ADL disability incidence and ensuing patterns of disability progression in a nationally representative sample of older Americans aged 80 and above. We exploit panel data over an 18-year period to paint a mathematical picture of functional decline among the oldest-old as they advance in age. We also explore the nexus between disability and chronic illnesses. This study is the first, to our knowledge, that longitudinally evaluates and compares ADL loss sequences for older adults with and without major chronic conditions. Three important findings, in particular, deserve comment.

First, our findings indicate that older adults who ever have any of the ‘big four’ NCDs are at higher risk of becoming functionally disabled than persons without such diseases. Disability incidence rates, across all ADL items, are higher for persons with major chronic diseases than for persons without such conditions. A widely-held assumption is that persons with major NCDs generally face higher risk of mortality. Our analysis reveals that it is critical to distinguish between persons with incident disability versus those without disability in this aspect. Specifically, we observe that only chronically ill older adults who are also functionally impaired are exposed to greater risk of death. The proportions of non-disabled older adults who died during the observation period is comparable between both subgroups.

Second, we show that disability onset is systematically earlier for older adults with major NCDs. In addition, and importantly, that their disability progression is compressed within a shorter timeframe. For the chronically ill, multiple disabilities strike almost at the same time and gaps between the onset of one disability and the next is small. In other words, this risk group face a steeper slope of functional decline as compared to their counterparts. This has profound implications. Chronic care delivery programs that seek to offer higher quality of care need to take into account that older patients may experience a loss of function or worsening of functional capabilities during their period of care, and care hours have to be changed accordingly to adapt to such dynamic realities. As a patient becomes afflicted with more ADL disabilities, chronic care can become more complex and expensive. This underscores the importance of consistent care for chronically ill persons for whom an interruption in care can lead to exacerbation, or even death.

Third, our analyses are informative on how ADL loss sequences compare between older Americans with and without major NCDs. We find two broad similarities. Regardless of chronic health status, the progression of functional loss is characterized by an early loss cluster and a late-loss cluster. The former comprises bathing, dressing, and walking, while the latter comprises toileting, transferring, and eating (items listed in no particular order). This separation of item clusters is consistent the finding in Fong & Feng [12] based on the Rasch scaling model. Another similarity is that bathing disability occurs first and the eating disability last in both risk groups – a finding that concurs with the ADL hierarchies derived in previous studies for geriatric populations in the U.S. and elsewhere [4–6, 10, 12]. One difference, however, in the two representative ADL orderings is that chronically ill persons are likely to lose functional capacities in a ‘BDW-TPE’ sequence whereas their counterparts tend to do so in a ‘BWD-PTE’ sequence. This subtle difference can be rationalized in part by the weak orderings observed in the early-loss cluster disabilities, and separately in the toileting and transferring disabilities, in our full sample as well as in some prior studies [3, 10].

## Conclusions

Our study of disability emphasizes that prevention of functional decline should target major noncommunicable diseases in older adults. In addition, disease management programs for chronically ill older adults should take a closer look at new interventions to help patients delay disability onset and optimize functional autonomy within emerging models of chronic care. The small gaps in onset ages within the cluster of early-loss disabilities is particularly worrisome as this suggests that these three disabilities tend to strike together. Consequently, older Americans and especially those with major chronic conditions who have difficulty with any one of these disabilities (e.g. bathing) are at high risk of developing the other two disabilities. Dependency in three or more ADLs, in turn, is associated with the need for long-term care and adverse outcomes such as nursing home admission [23, 25, 26].

This study has limitations. First, the AHEAD measures of ADLs are self-reported, yet normative perceptions of “having difficulty” with a particular task may vary across individual respondents. For example, some studies contend that individuals are more likely to report the having difficulty with self-care tasks if they have access to caregivers [27]. Second, sample shrinkage over the follow-up period is another limitation. Mortality tends to be a problem in most studies focusing on the oldest adults, and in our case, the low rate of attrition or being lost to follow-up (1.8%) provides some reassurance. Future research using richer longitudinal data can investigate further how patterns in disability onset vary by specific diseases and whether such patterns are modifiable depending on individuals’ health behaviour, social supports and other factors in the environment. Further work is also needed to develop prevention strategies to delay onset of ADL disabilities and interventions to meet the needs of older people as these disabilities occur.

## Data Availability

The datasets analysed in the current study are publicly available in the Health and Retirement Study repository. The data products are available without cost to registered users. More information can be found at (http://hrsonline.isr.umich.edu).

## Abbreviations

ADL: Activities of daily living
AHEAD: Asset and Health Dynamics of the Oldest Old
CVD: Cardiovascular diseases
IRT: Item response theory
NCD: Noncommunicable disease
